# Birth plan presentation to hospitals and its relation to obstetric outcomes and selected pain relief methods during childbirth

**DOI:** 10.1186/s12884-021-03739-z

**Published:** 2021-04-01

**Authors:** Encarnación López-Gimeno, Gemma Falguera-Puig, Mª. Mercedes Vicente-Hernández, Meritxell Angelet, Griselda Vázquez Garreta, Gloria Seguranyes

**Affiliations:** 1 Sexual and Reproductive Healthcare Services (ASSIR), Catalan Health Institute (ICS), Gran Via de les Corts Catalans, 587, 08006 Barcelona, Spain; 2 Cap Sant Felix, Carretera de Barcelona 473, 08206 Sabadell Barcelona, Spain; 3 Sexual and Reproductive Healthcare Services (ASSIR), Directorate of Primary Healthcare Service - North Metropolitan Area, Catalan Health Institute (ICS), Barcelona, Spain; 4 Faculty of Medicine and Health Sciences, Nursing School, Campus Bellvitge, Universitat de Barcelona, L’Hospitalet del Llobregat, Barcelona, Spain

**Keywords:** Birth plan, Midwife, Pain relief, Obstetric labour

## Abstract

**Background:**

The information on birth plan (BP) usage in Spanish hospitals is scant.

**Aim:**

To identify the percentage of pregnant women presenting a BP at five hospitals in Spain, the reasons why some women failed to do so and how BP presentation relates to obstetric outcomes and selected pain relief methods.

**Methods:**

In this descriptive, multi-centre study, data were retrospectively collected. During the postpartum visits at primary healthcare centres in various health districts in Barcelona (Catalonia, Spain), a data collection sheet about obstetric outcomes and analgesia was administered to 432 mothers who had completed a BP during their pregnancies. The main outcome was the rate of BP presentation to the hospital. The sociodemographic and obstetric characteristics and pain relief measures were compared to identify any differences between mothers who presented a BP and those who did not.

**Results:**

A total of 422 (99.7%) women were studied; 51.2% of women (95% confidence interval (CI): 46.4–55.9) had presented a BP. The main reason for not presenting a BP was because the hospital midwives did not request them (61.2%). No differences were observed in BP presentation according to age, the country of origin, education, employment or hospital. Mothers who presented a BP were more likely to start breastfeeding in the birthing room (82.4% vs. 73.3%; *p* = 0.024). Epidural analgesia was the most common method used for pain relief (88.9%), and women who presented a BP attempted to use concomitant non-pharmacological methods more often (50.5% vs. 38.8%; *p* = 0.012).

**Conclusion:**

Almost half of the mothers failed to present a BP, usually because midwives did not request it.

## Background

Birth plans (BPs) were developed to improve informed decision-making by mothers and improve communication and cooperation between healthcare providers and users [1].

However, the format of these BPs and the approach to BP use are still a subject of debate [1, 2].

In essence, BPs are a tool that can enhance communication between women and healthcare professionals. Furthermore, the preparation of a BP is an educational activity, as it increases mothers’ knowledge regarding childbirth [3, 4], and professionals should adopt educational strategies during prenatal care and childbirth to facilitate BP conversations with mothers and help them make decisions related to the birth of their child [5, 6].

In 2012, the Spanish Ministry of Health published a framework document on BPs [7], and in the autonomous region of Catalonia, each maternity and children’s hospital developed a BP template in conjunction with primary healthcare centres that provide sexual and reproductive healthcare services (ASSIR).

In our setting, in the Public Health Service**,** women are given prenatal care by midwives in ASSIR health centres, and it is recommended that midwives discuss the options listed in the BP with women around weeks 29–30 of pregnancy and ask them to bring the BP on the day of birth. Birth takes place in hospitals, where midwives and obstetricians provide health care according to the level of obstetric risk [8].

Two studies in Catalonia, Spain, have reported that 86.9 to 98.8% of mothers receive BP information from midwives during prenatal care [9, 10]. In Spain, there are no comprehensive data on the prevalence of BP presentation to hospitals. In fact, the percentage of women presenting a BP to hospitals varies from 2.8 to 69.6% [10, 11]. Only a few studies in the USA and Europe describe how many women present a BP when they are admitted to the hospital, with figures varying from 12% to 39.8 [12–14].

Likewise, studies have examined whether BPs influence obstetric results and the use of methods for pain relief in childbirth. There is some controversy in the literature. BPs are not related to an increased number of vaginal births [15]; however, Asfhar showed an association of BPs with vaginal birth [12]. The results for caesarean delivery are different; while one study showed a reduction in the number of caesarean deliveries [16], another study found no reduction [17].

Studies have investigated the relationship of BPs with pharmacological pain relief methods [16, 18] but not with the use of non-pharmacological methods or their combination. Concerning epidural analgesia, we found opposing results: one study showed less use of epidural analgesia in women using a BP [17], whereas the Hadar study presented an increase in epidural use in women using a BP [16].

Among women who receive BP counselling during prenatal check-ups, the prevalence of BP presentation to the hospital is unknown. The aim of this study was to identify the prevalence of BP presentation to five hospitals in Catalonia and to learn the reasons why mothers failed to provide a BP. An additional aim was to determine whether the women’s characteristics or the hospital had any influence on their decisions and to determine whether BP presentation to the hospital is related to the obstetric outcomes and to the pain relief methods chosen.

## Methods

### Study design and participants

The study was based on a descriptive, multicentre design with retrospective data collection, with the participation of five ASSIR units in primary care centres located in Catalonia.

Approximately 6094 women received antenatal and postpartum care during 2017 at these five ASSIR units of the Catalan Health Institute. The study included 432 women.

The sample size at each centre was proportional to the number of births in the reference hospitals. Granmo software (version 7.12, IMIM, Barcelona, Spain) was used to calculate the sample size. It was estimated that to achieve a population percentage of 50%, with a 95% confidence interval, a precision of ±5% points and a replacement level of 15%, 422 women should be included [19].

Participants were recruited between March and December 2017 at the postpartum appointment in the healthcare centre, at which time midwives informed the women of the aim of the study and invited them to participate. Women who wished to participate gave their written consent. Mothers who had given birth in the reference hospital, completed a BP during pregnancy, received prenatal care and attended the six-week postpartum appointment with midwives at the primary care study sites were eligible to be included in the study. Mothers under age 18 years, those who had language difficulties or those who had experienced a perinatal death were excluded from the study. Once enrolled, midwives collected information from mothers during postpartum care in the ASSIR.

A data collection sheet was designed to collect demographic and obstetric information on the mothers and obstetric and neonatal outcomes for the infants, as well as information on whether or not the woman had presented a BP and, if not, the reason why. A pilot test was conducted previously with 20 mothers to determine the feasibility of data collection and to identify and correct any aspects that could be improved. These data were not included in the analysis. The study was approved by the Clinical Research and Ethics Committee of the University Institute for Research in Primary Care, and all participants gave written informed consent.

### Outcome measures

#### Prevalence of BP presentation

The primary variables included BP presentation (yes/no) and women’s reasons for non-presentation (I didn’t think it was necessary/I forgot it/the midwives didn’t ask me for it/other).

#### Obstetric outcomes

Four variables were studied: the type of birth (spontaneous/assisted vaginal birth/caesarean section); episiotomy (yes/no); the initiation of skin-to-skin contact (yes/no) and the initiation of breastfeeding in the birthing room (yes/no).

#### Pain relief methods

Pain relief methods were divided into three categories (pharmacological/non-pharmacological/pharmacological plus non-pharmacological). In the case of pharmacological pain relief, the methods were classified as epidural analgesia, local anaesthesia, general anaesthesia and/or nitrous oxide, and in the case of non-pharmacological pain relief, the methods were classified as relaxation techniques, breathing techniques, massage, water use, local heat, the use of a birthing ball, or others (acupuncture, aromatherapy, homeopathy, Bach flowers). When both pharmacological and non-pharmacological methods were used, the method was classified as a combination method.

#### Co-variables

The study collected additional information on the demographics and maternal health of the women, specifically their age, country of origin (Spain/other), education (primary school or less/high school/university), employment (yes/no), previous birth(s) (yes/no) and obstetric risk (low/high).

### Statistical analysis

The data were entered into an SPSS 24.0 software database and were accessible only to the study investigators. Descriptive data are expressed as numbers and percentages and as the mean ± standard deviation (SD). The 95% confidence intervals (Cis) for the primary outcome measures were calculated.

Differences in sociodemographic and obstetric profiles and in the presentation of a BP to the hospital were analysed using the chi-squared test or Fisher’s exact test, as appropriate. All quantitative variables were compared using Student’s *t* test.

A multivariate logistic regression analysis was performed to identify variables related to BP presentation to the hospital (the dependent variable). The independent variables were age, the country of origin, education, employment and hospital. The adjusted odds ratio (OR) and 95% CI were determined. A *p* value < 0.05 was considered statistically significant.

## Results

A total of 432 women were studied. However, ten were excluded due to incorrect completion of the data collection sheet, yielding a total of 422 (97.7%) women for the final analysis.

Table 1 lists the sociodemographic and maternal health characteristics of the mothers according to BP presentation to hospitals. The mean age of the sample was 31.4 years (SD = 5.1), 71.3% were Spanish, 47.7% had a high school level of education, and 72.3% were employed. A total of 54.7% were primiparous, and 72.7% had a low obstetric risk level. There were no differences according to the presentation of a BP.

**Table 1:** Demographic and maternal health characteristics of the women according to whether a birth plan was presented

		Birth plan presented		
	Total	Yes	No	
	*N* = 422	*n* = 216 (51.2)	*n* = 206 (48.8)	*p*
**Age, mean (SD)**	31.4 (5.1)	31.2 (5.2)	31.6 (4.9)	0.406^a^
**Country of origin, n (%)**				
Spain	301 (71.3)	158 (73.1)	143 (69.4)	0.397^b^
Other	121 (28.7)	58 (26.9)	63 (30.6)	
**Education, n (%)**				
Primary school or less	82 (19.4)	37 (17.1)	45 (21.8)	0.332^b^
High school	201 (47.7)	102 (47.2)	99 (48.1)	
University	139 (32.9)	77 (35.7)	62 (30.1)	
**Employment, n (%)**				
Yes	305 (72.3)	156 (72.2)	149 (72.3)	0.980^b^
No	117 (27.7)	60 (27.8)	57 (27.7)	
**Previous birth(s), n (%)**				
Yes	191 (45.3)	95 (44)	96 (46.6)	0.589^b^
No	231 (54.7)	121 (56)	110 (53.4)	
**Obstetric risk level, n (%)**				
Low	307 (72.7)	161 (74.5)	146 (70.9)	0.398^b^
High	115 (27.3)	55 (25.5)	60 (29.1)	

Data are presented as n (%) or the mean (SD); ^a^Student’s t test; ^b^chi-squared test; *p* = *p* value

The presentation of a BP (yes/no) and the reasons for failing to do so are shown in Table 2 according to the hospital. The percentage of mothers presenting a BP to the hospital was 51.2% (95% CI, 46.4 to 55.9). Women receiving care in hospital #3 presented a BP less frequently (*n* = 68; 61.3%), and significant differences were observed among the hospitals (*p* = 0.014). An analysis of the reasons why 206 women (48.8%) did not present a BP to the hospital showed that the main reason was “the midwives didn’t ask me for it” (*n* = 126; 61.2%), and no differences in reasons were found among women.

**Table 2:** Birth plan presentation by hospital and reasons for non-presentation

		Hospital					
		#1	#2	#3	#4	#5	
	Total	n (%)	n (%)	n (%)	n (%)	n (%)	*p*
**Birth plan was presented**	*N* = 422 (%)	50 (11.8)	59 (14)	111 (26.3)	91 (21.6)	111 (26.3)	0.014^a^
Yes	216 (51.2)	26 (52)	28 (47.5)	43 (38.7)	56 (61.5) *	63 (56.8)	
No	206 (48.8)	24 (48)	31 (52.5)	68 (61.3)	35 (38.5)	48 (43.2)	
**Women’s reasons for not presenting a birth plan**	*N* = 206 (%)	24 (11.7)	31 (15)	68 (33)	35 (17)	48 (23.3)	0.19^b^
I didn’t think it was necessary	11 (5.3)	3 (12.5)	0 (0)	3 (4.4)	3 (8.6)	2 (4.2)	
I forgot	28 (13.6)	2 (8.3)	8 (25.8)	9 (13.2)	7 (20)	2 (4.2)	
Nurse-midwives didn’t ask me for it	126 (61.2)	14 (58.3)	20 (64.5)	47 (69.1)	18 (51.4)	27 (56.2)	
Other causes	41 (19.9)	5 (20.9)	3 (9.7)	9 (13.3)	7 (20)	17 (35.4)	

Data are expressed as n (%); ^a^chi-squared test; ^b^Fisher’s exact test; *p* = p value; *p < 0.05

The multivariate model used to study the relationship between the independent variables and BP presentation (Table 3) found that birth in a specific hospital did not influence the percentage of women who presented a BP. No other independent variables influenced BP presentation.

**Table 3:** Multivariate analysis of factors related to birth plan presentation to the hospital

Birth plan presented	aOR (95% CI)	*p*
**Age**	0.97 (0.92–1.01)	0.118
**Country of origin**		
Other	Ref	
Spain	1.27 (0.80–2)	0.314
**Education**		
Primary school or less	Ref	
High school	1.30 (0.76–2.23)	0.555
University	1.38 (0.73–2.6)	0.336
**Employment**		
No	Ref	
Yes	0.92 (0.58–1.46)	0.732
**Hospital**		
Hospital #1	Ref	
Hospital #2	0.96 (0.44–2.08)	0.910
Hospital #3	0.58 (0.30–1.16)	0.122
Hospital #4	1.45 (0.71–2.94)	0.309
Hospital #5	1.39 (0.66–2.92)	0.380

aOR* Adjusted odds ratio; *95% CI* 95% Confidence interval; *p* = *p* value

Table 4 lists the obstetric outcomes, with 66.9% of mothers having spontaneous births. The results of the type of labour, episiotomy, and initiation of skin-to-skin contact were similar in women who did and in those who did not present a BP to the hospital. Breastfeeding was started in the birthing room by a significantly higher number of women (82.4% vs. 73.3%; *p* = 0.024) who did present a BP.

**Table 4:** Birth plan presentation according to obstetric outcomes

		Birth plan presented		
	Total n (%)	Yes n (%)	No n (%)	*p*
	*N* = 422	*n* = 216 (51.2)	*n* = 206 (48.8)	
**Type of labour**				
Spontaneous birth	282 (66.9)	146 (67.6)	136 (66)	0.377^a^
Assisted vaginal birth	74 (17.5)	41 (19)	33 (16)	
Caesarean birth	66 (15.6)	29 (13.4)	37 (18)	
**Episiotomy**				
Yes	155 (36.7)	81 (37.5)	74 (35.9)	0.737^a^
No	267 (63.3)	135 (62.5)	132 (64.1)	
**Initiation of skin-to-skin contact**				
Yes	381 (90.2)	199 (92.1)	182 (88.4)	0.19^a^
No	41 (9.8)	17 (7.9)	24 (11.6)	
**Initiation of breastfeeding in birthing room**				
Yes	329 (78)	178 (82.4)	151 (73.3) *	0.024^a^
No	93 (22)	38 (17.6)	55 (26.7)	

Data are expressed as n (%); ^a^chi-squared test; *p* = p value; *p < 0.05

The different methods of pain relief used by women are presented in Table 5. Nearly all mothers (*n* = 386; 91.2%) were given some pharmacological measure of pain relief, with epidural analgesia being the most common (*n* = 375; 88.9%). The use of epidural analgesia was similar in women who did and in those who did not present a BP (86.1% vs 91.7%). In fact, 36% of women combined pharmacological and non-pharmacological methods. Women who presented a BP used non-pharmacological methods more often than women who did not present a BP (50.5% vs. 38.8%; *p* = 0.012). The following non-pharmacological methods were more likely to be used by women who presented a BP than by those who did not, with all differences being statistically significant: relaxation techniques (21.7% vs. 12.6%), breathing techniques (40.7% vs. 28.2%), the use of water (13.4% vs. 7.3%) and the use of local heat (15.7% vs. 7.3%).

**Table 5:** Birth plan presentation according to the use of pain relief methods

		Birth plan presented		
**Pain relief method**	**Total** n (%)	**Yes** n (%)	**No** n (%)	*p*
	*N* = 422 (%)	*n* = 216 (51.2)	*n* = 206 (48.8)	
**Pharmacological**				
Yes	386 (91.2)	195 (90.3)	191 (92.7)	0.370^a^
No	36 (8.8)	21 (9.7)	15 (7.3)	
**Epidural analgesia**				
Yes	375 (88.9)	186 (86.1)	189 (91.7)	0.066^a^
No	47 (11.1)	30 (13.9)	17 (8.3)	
**Local anaesthesia**				
Yes	14 (3.3)	11 (5) *	3 (1.5)	0.037^b^
No	408 (96.7)	205 (95)	203 (98.5)	
**Nitrous oxide**				
Yes	2 (0.5)	2 (0.9)	0 (0)	0.166^b^
No	420 (99.5)	214 (99.1)	206 (100)	
**General anaesthesia**				
Yes	4 (1)	3 (1.4)	1 (0.5)	0.338^b^
No	418 (99)	213 (98.6)	205 (99.5)	
**Non-pharmacological**				
Yes	189 (44.8)	109 (50.5) *	80 (38.8)	0.012^a^
No	233 (55.2)	107 (49.5)	126 (61.2)	
**Relaxation techniques**				
Yes	73 (17.3)	47 (21.7) *	26 (12.6)	0.013^a^
No	349 (82.7)	169 (78.3)	180 (87.4)	
**Breathing techniques**				
Yes	146 (34.6)	88 (40.7) *	58 (28.2)	0.007^a^
No	276 (65.4)	128 (59.3)	148 (71.8)	
**Massage**				
Yes	59 (14)	35 (16.2)	24 (11.7)	0.178^a^
No	363 (86)	181 (83.8)	182 (88.3)	
**Use of water**				
Yes	44 (10.4)	29 (13.4) *	15 (7.3)	0.039^a^
No	378 (89.6)	187 (86.6)	191 (92.7)	
**Use of local heat**				
Yes	49 (11.6)	34 (15.7) *	15 (7.3)	0.007^a^
No	373 (88.4)	182 (84.3)	191 (92.7)	
**Use of a birthing ball**				
Yes	32 (7.6)	19 (8.8)	13 (6.3)	0.335^a^
No	390 (92.4)	197 (91.2)	193 (93.7)	
**Other methods**^**#**^				
Yes	8 (1.9)	5 (2.3)	3 (1.5)	0.518^b^
No	414 (98.1)	211 (97.7)	203 (98.5)	
**Pharmacological + non-pharmacological**				
Yes	152 (36)	87 (40.3)	65 (31.5)	0.062^a^
No	270 (64)	129 (59.7)	141 (68.5)	

Data are expressed as n (%); ^a^chi-squared test; ^b^Fisher’s test; *p* = p value; *p < 0.05; ^*#*^other methods include acupuncture, aromatherapy, homeopathy, and Bach flowers

## Discussion

The present study assessed the prevalence of BP presentation to five hospitals in Catalonia and its relation to obstetric outcomes and pain relief methods during childbirth.

The total prevalence of BP presentation to hospitals was 51.2%, a lower result than the 69.6% reported in another study conducted in Catalonia [10]. One explanation for this difference may be that our study was conducted in five different healthcare centres, whereas the earlier study was conducted in a single healthcare centre. The prevalence found in our study is also higher than that found in other studies carried out in the USA [12] and the Netherlands [13].

Mothers reported that they failed to provide a BP because “the midwives did not ask me for it” or “I forgot”. Thus, two-thirds of the women who did not present a BP to the hospital stated that it was not requested by any healthcare professional. It is likely that the reason for the non-proactive behaviour related to the BP being requested by these healthcare professionals could be due to the perception that these women would have worse obstetric outcomes, as shown in other studies [20, 21]. It is also possible that some midwives believed that BPs might make mothers inflexible and unwilling to change their plan, giving them a false feeling of control or creating unrealistic expectations of birth, leading to disappointment if the BP was not fulfilled [3]. Likewise, some midwives have expressed that the use of BPs produces a feeling of judgment and pressure from the mothers [22]. In contrast, professionals who fail to ask for a BP may cause a sense of disappointment in women at the time of birth, as these women may feel that the preferences stated in their BPs were not considered and there was no ensuing discussion of their options [23].

Conversely, only 5.3% of mothers considered that “it wasn’t necessary” to provide a BP. Several studies have analysed the opinions of mothers and how they perceive BPs. According to the literature, mothers who did not use BPs reported that they trusted the professionals’ experience or that BPs were not useful because of the situations that could arise during birth [23, 24].

Similar to some studies [16, 17], we found that the presentation of a BP was not related to the vaginal delivery or the need for an episiotomy. The percentage of women who initiated skin-to-skin contact in our study was high in both groups and differs from that found in a study carried out in Spain, which was 60.4% among women with BPs [11]. However, the presentation of a BP was related to a high initiation of breastfeeding in the delivery room, but this finding differs from that of another study that found a similar percentage of women initiating breastfeeding in those who did and did not present a BP [11]. Our finding could be because the option of breastfeeding is one of the most discussed issues in BPs [25] and because the initiation of breastfeeding in the delivery room is one of the most common requests made by women in BPs [26, 27].

Epidural analgesia was the pain relief method used most often, and the frequency of the use of epidural analgesia was very high because epidural analgesia is the most frequent pharmacological method provided in the hospitals under study. This high prevalence is consistent with that reported in two other studies [11, 16]. The use of epidural analgesia was similar in the two groups, as in the Hidalgo study [15]. However, in the Hadar study [16], the use of epidural analgesia was higher in women with BPs, and in the Asfhar study, the use of epidurals was lower in women without a BP [17].

Therefore, the high use of epidural analgesia may be because epidural analgesia is considered the gold-standard pain relief method [28], and it is the most preferred method expressed by women in BPs in some studies [27, 29]. Additionally, some factors may influence a woman’s choice to undergo an epidural, such as her pain threshold, her ability to cope with pain, the timing of the epidural and the length of labour [30].

During this particular study, only one of the hospitals started providing nitrous oxide analgesia, which is the reason why we found the percentage of its use to be low.

One-third of the women who presented a BP to the hospital received both pharmacological and non-pharmacological measures to relieve pain. According to the Larkin study [31], mothers considered the availability of pain relief measures and the possibility of combining pharmacological and non-pharmacological methods as priorities of their childbirth experience. Furthermore, they stated “most women did not want to be typified as wanting the dichotomy of ‘all natural’ or ‘all technology’ births but wanted ‘the best of both worlds’.

Additionally, half of the women who presented a BP to the hospital used different non-pharmacological pain relief methods, such as relaxation techniques, breathing techniques, the use of water and the use of local heat. These women did so probably as a first step in relieving pain during labour, even if most of these women subsequently used epidural analgesia. Gallo et al. [32] reported the use of different non-pharmacological techniques to reduce or delay the use of pharmacological analgesia.

In our study, the rates of the use of non-pharmacological methods were low compared to those reported in other studies [33, 34]. To our knowledge, the relationship between BP presentation and the use of non-pharmacological methods for pain relief has not been studied. The use of these methods requires continuous care by the midwife [35], and in Spain, the proportion of midwives in hospitals is low [36], which makes continuous and individualized intrapartum care, as recommended by the World Health Organization, difficult [37]. Likewise, the limited training of midwives in these methods and in alternative therapies could also act as a barrier to their use [38].

Despite this, the women who presented BPs used these methods in a greater proportion. This finding may be due to the effect that the presentation of a BP has on professionals, in that they are more attentive to the preferences of women [3].

### Implications for clinical practice

Hospital midwives need to routinely ask women on admission for their BPs and discuss their content. Maternity hospitals must provide pharmacological analgesia as well as different non-pharmacological methods because women want to use them for pain relief during labour.

### Strengths and limitations

To the best of our knowledge, this is the first study to describe the relationship between the presentation of a BP and the use of various pain relief methods. The information was collected within the first 6 weeks after birth, which may be a limitation, as there may be a memory bias in women [39]. Another possible limitation is the exclusion of women with difficulties understanding the language; therefore, it is not known whether these women used a BP. Finally, another possible limitation is that the information was collected from the midwives of healthcare centres, as no unified hospital record system on BP usage has been developed.

## Conclusion

This exploratory study showed that one of every two mothers did not provide a BP to the hospital, with more than half of these women stating that they failed to do so because midwives did not ask for a BP. No independent demographic characteristics were found to be associated with BP presentation. Mothers who presented a BP to the hospital were more likely to commence breastfeeding in the birthing room. Most of the women received epidural analgesia, and half of the women who presented a BP to the hospital used non-pharmacological measures to relieve pain.

## Data Availability

The datasets used and analysed during the current study are available from the corresponding author upon reasonable request.

## Abbreviations

BP: Birth plan
ASSIR: Sexual and reproductive healthcare services
CI: Confidence interval
